# Secreted Extracellular Vesicle Molecular Cargo as a Novel Liquid Biopsy Diagnostics of Central Nervous System Diseases

**DOI:** 10.3390/ijms22063267

**Published:** 2021-03-23

**Authors:** Sara Monteiro-Reis, Carina Carvalho-Maia, Genevieve Bart, Seppo J. Vainio, Juliana Pedro, Eunice R. Silva, Goreti Sales, Rui Henrique, Carmen Jerónimo

**Affiliations:** 1 Cancer Biology & Epigenetics Group, Research Center, Portuguese Oncology Institute of Porto (CI-IPOP), R. Dr. António Bernardino de Almeida, 4200-072 Porto, Portugal; sara.raquel.reis@ipoporto.min-saude.pt (S.M.-R.); carina.carvalho.maia@ipoporto.min-saude.pt (C.C.-M.); rmhenrique@icbas.up.pt (R.H.); 2 Biobank, Department of Pathology, Portuguese Oncology Institute of Porto (IPOP), R. Dr. António Bernardino de Almeida, 4200-072 Porto, Portugal; 3 Disease Networks Research Unit, Laboratory of Developmental Biology, Faculty of Biochemistry and Molecular Medicine, Infotech Oulu, Kvantum Institute, University of Oulu, 90570 Oulu, Finland; genevieve.Bart@oulu.fi (G.B.); seppo.vainio@oulu.fi (S.J.V.); 4 Psychology Service, Portuguese Oncology Institute of Porto (IPOP), R. Dr. António Bernardino de Almeida, 4200-072 Porto, Portugal; juliana.batista.pedro@ipoporto.min-saude.pt (J.P.); esilva@ipoporto.min-saude.pt (E.R.S.); 5 BioMark Sensor Research/Centre of Biological Engineering of Minho University, Campus de Gualtar, 4710-057 Braga, Portugal; goreti.sales@gmail.com; 6 Department of Chemical Engineering, Faculty of Sciences and Technology, University of Coimbra, Rua Sílvio Lima, 3030-790 Coimbra, Portugal; 7 Department of Pathology and Molecular Immunology, Institute of Biomedical Sciences Abel Salazar, University of Porto (ICBAS-UP), Rua Jorge Viterbo Ferreira 228, 4050-513 Porto, Portugal

**Keywords:** extracellular vesicles, central nervous system, biomarkers

## Abstract

Secreted extracellular vesicles (EVs) are heterogeneous cell-derived membranous granules which carry a large diversity of molecules and participate in intercellular communication by transferring these molecules to target cells by endocytosis. In the last decade, EVs’ role in several pathological conditions, from etiology to disease progression or therapy evasion, has been consolidated, including in central nervous system (CNS)-related disorders. For this review, we performed a systematic search of original works published, reporting the presence of molecular components expressed in the CNS via EVs, which have been purified from plasma, serum or cerebrospinal fluid. Our aim is to provide a list of molecular EV components that have been identified from both nonpathological conditions and the most common CNS-related disorders. We discuss the methods used to isolate and enrich EVs from specific CNS-cells and the relevance of its components in each disease context.

## 1. Introduction

Extracellular vesicles (EVs) are cell-derived, membrane-bound structures, secreted into the extracellular environment by most, if not all, cell types. There is mounting evidence that EVs take part in control of normal physiological processes, in homeostasis control and in cell-to-cell communication within tissues and organs. Based on development of the EV field, different types of vesicles have been described, and include apoptotic bodies which range in size from 50 to 5000 nm, microvesicles (50–1000 nm), exosomes (40–200 nm) and the more recently recognized exomeres [1,2,3,4].

The EVs have started to attract attention also as potential disease biomarkers and as new ways to study the control of cell biology. They seem to offer diagnostic capacity of organ dysfunctions via the body fluids as the components of the “liquid biopsy” type of sample. For example, solid tumors exhibit the elusive nature of transformed cells. They grow into organs but can metastasize to distant locations. The original tumor cells and their metastases secrete EVs and this has offered a promising way to diagnose cancers from its inception [5]. By studying the detailed molecular content of the cancer cell secreted EVs, tumor specific signatures may be defined and information about the changes in the associated biological processes gained.

In central nervous system (CNS) diseases, such as Alzheimer’s disease, Parkinson’s disease, multiple sclerosis, amyotrophic lateral sclerosis, gliomas, traumatic brain injury, stroke, schizophrenia and major depressive disorders, neuroscientists and neurologists cannot easily access the diseased tissues for diagnostic purposes. If brain biopsy is to be taken, it might not be representative of the common neurological disorder. Therefore, if the EVs that would be released from the CNS to systemic circulation could be identified reliably, this would provide a breakthrough to advance diagnostics of neurological diseases [6].

One of the challenges when working with EVs, particularly for CNS and CNS-related pathological conditions, is to ensure that the CNS-derived EVs can be isolated from the biological fluids (e.g., plasma (containing blood clotting agents), serum (without blood clotting agents) or cerebrospinal fluid (CSF)) and that these indeed would have originated from neural cells. Since EVs also contain the HLA-associated, self-depicting peptides and wealth of other types of cargo proteins of donor cells, such EV molecules may serve to identify specific organ level signatures, thus portraying the cell of origin. Since the EVs also have the ability to cross the blood–brain barrier (BBB) this raises the possibility that EV molecular content in biological fluids may reflect certain neuronal processes, as well [7]. Currently a relatively large body of literature documents the importance of EVs, but none has yet systematically summarized EVs molecular content in association with CNS and common CNS-related diseases. In this review, we provide a catalogue of CNS-derived EVs’ molecular cargo, both in nonpathological and selected pathological conditions.

## 2. Methods

**Figure 1:**
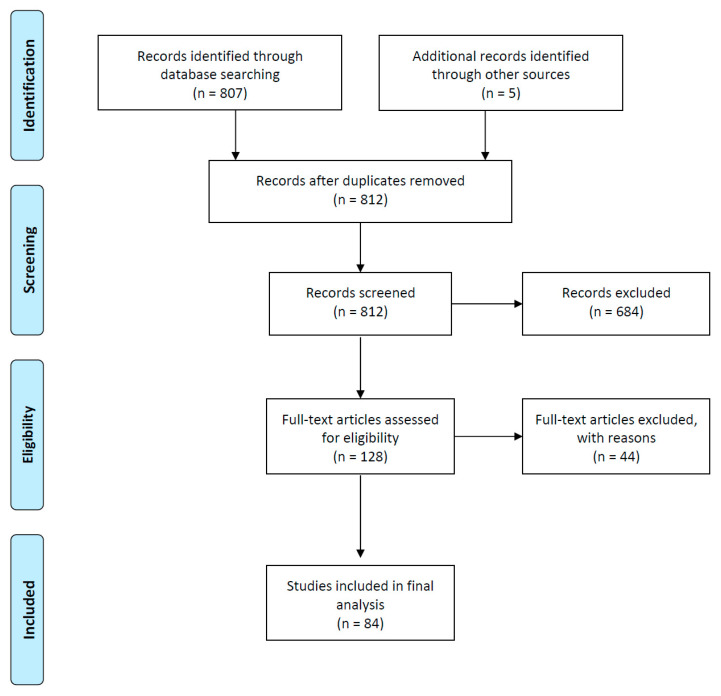
Flow diagram representing a summary of the conducted methodology for this review.

A PubMed search was conducted, with the query (extracellular vesicles OR exosomes OR microvesicles) AND (markers OR biomarkers) AND (brain OR central nervous system), with no time interval restraints. Only original studies written in English were considered. The retrieved records from the search were collected into the reference manager Endnote. All abstracts were critically assessed to select only those providing meaningful information related to the topic. Only studies using human cerebrospinal fluid (CSF), serum or plasma samples were included. A flow diagram with a summary of the methodology is provided in Figure 1.

## 3. Methods and Factors for Isolation of Central Nervous System Extracellular Vesicles in Liquid Biopsies

Currently, as EVs are known to be able to cross the BBB, they are considered as potential biomarkers for monitoring CNS disorders as well as specific treatment responses. Of particular interest is the ability to characterize EVs based on their source, which may provide insight into the target disease. Although the majority of EVs research is performed through cell culture, the number of reports aiming at identifying biological fluids’ EVs originated in different cell types is rising. Multiple methods (including ultracentrifugation, immunomagnetic beads, and size exclusion chromatography) can be used to separate mixed EVs (from multiple cell types) from different biological fluids [8]. This step may be taken further by using cell-specific protein markers to enrich for EVs of specific cell types from a mixed population of vesicles.

Specifically, EVs from the CNS are secreted from almost all cell types, including various types of neurons, astrocytes, oligodendrocytes, microglia and Schwann cells, and endothelial cells. EVs carry a variety of molecules related to neuronal function and neurotransmission in the brain. This contributes to the communication between nerve cells (for example, neuron–glia interaction), synaptic plasticity, and neuron development. Interestingly, Fiandaca et al. developed an immunoprecipitation-based method to isolate EVs rich in neuron sources from blood. This technology has been also used by others and consists of two steps: a preliminary separation of total EVs from plasma or serum samples using commercially available polymers for high-throughput particle precipitation, and immunoprecipitation with biotinylated antibodies against the neuron surface marker neuronal markers L1 cell adhesion molecule (L1CAM, CD171) [9]. Furthermore, L1CAM and the GluR2/3 subunits of glutamate receptors have been used for the identification of EVs that may be released from developing and mature hippocampal neurons [10,11]. Hence, using this method, EVs of neuronal origin may be enriched and evaluated based on proteins, lipids and nucleic acids.

**Figure 2:**
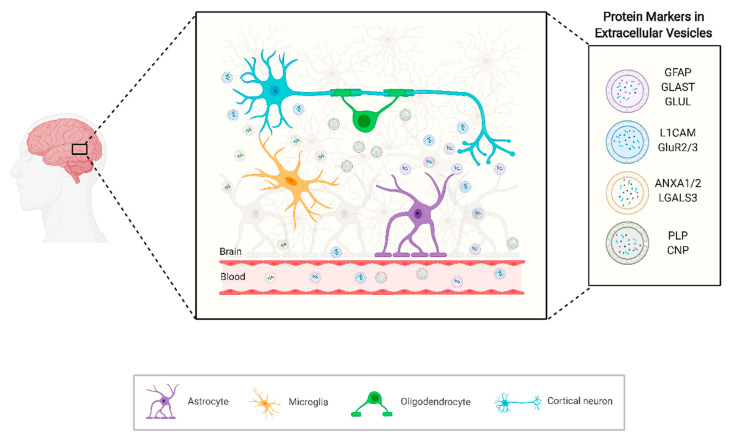
Schematic representation of the brain microenvironment, its main cellular components and how the brain–blood-barrier permeability allows for extracellular vesicles to reach circulation. Created with BioRender.com.

Although neuron-derived EVs are a common target in several studies, researchers are also interested in isolating EVs originating from other CNS cells, aiming also to translate via the EVs the origin of CNS-related diseases. The above-mentioned methodology can also be used to enrich for EVs from other cellular origins with antibodies against specific cell surface components. For example, for astrocytic-derived EVs enrichment, glial fibrillary acidic protein (GFAP), glutamine aspartate transporter (GLAST), and glutamine synthetase (GLUL) have been suggested to be specific enough to obtain CNS related EVs [12]. Additionally, for EVs originated in oligodendrocytes, myelin proteolipid protein (PLP) and 2′,3′-cyclic nucleotide 3′-phosphodiesterase (CNP) have been proposed as specific enough to identify the cell from which the EVs derived [13] (Figure 2).

The application of specific immunoaffinity-based methodology for the isolation of CNS-related EVs in biological fluids, applied to different CNS diseases research, has led to promising results [9,14]. Once the enriched fraction of EVs is obtained, either surface or cargo content can be analyzed using different approaches according to the molecular target of interest, such as proteomics (e.g., mass spectrometry, Western blot or enzyme-linked immunosorbent assays) and/or transcriptomics methodologies (e.g., high-throughput RNA sequencing or RT-qPCR). This opens a new avenue for research, using CNS-derived EVs as vehicles for disease monitoring.

## 4. Central Nervous System Disease-Related Biomarkers in Extracellular Vesicles

**Figure 3:**
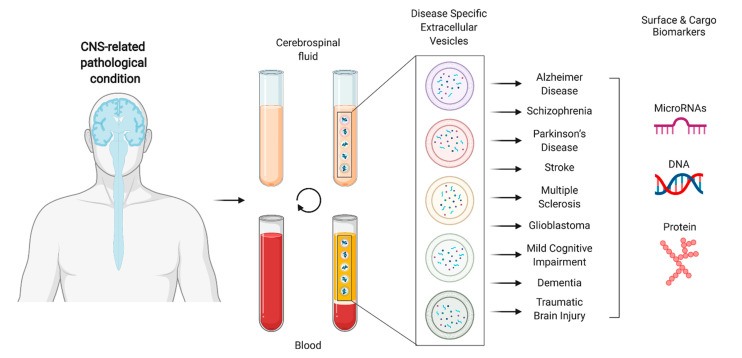
Schematic representation of the use of extracellular vesicles in liquid biopsies for identification of specific central nervous system (CNS)-related pathological conditions. Created with BioRender.com (accessed on 20 March 2021).

CNS-related disorders, including neurodegenerative diseases, such as Alzheimer’s (AD) and Parkinson’s (PD) diseases, malignant tumors, such as gliomas, and psychiatric disorders, are very different pathological conditions, but share common features: anatomical origin and difficult early diagnosis. The existing liquid biopsies and imaging biomarkers are rather imperfect, because their dynamic range does not cover the entire course of the disease, and their classification accuracy is lower than the level accepted for clinical practice, thus, precluding their routine implementation. Thus, the use of CNS-enriched EVs and their molecular content as biomarkers for these specific pathological conditions may revolutionize the clinical management of these patients. Indeed, a growing number of studies within this field have been published in the last few years. To simplify, a summary of the main publications retrieved from the database query can be found in Table 1 and Table 2, referring to either cargo or surface molecules, respectively [9,12,15,16,17,18,19,20,21,22,23,24,25,26,27,28,29,30,31,32,33,34,35,36,37,38,39,40,41,42,43,44,45,46,47,48,49,50,51,52,53,54,55,56,57,58,59,60,61,62,63,64,65,66,67,68,69,70,71,72,73,74,75,76,77,78,79,80,81,82,83,84,85,86,87,88,89,90,91,92,93,94,95,96,97] (Figure 3).

Alzheimer’s disease (AD) is neurodegenerative disorder characterized by memory loss and behavioral changes. EVs have been studied as a potential diagnostic marker for AD. This disease has a complex progression including early development of neuronal dysplasia, angiogenic changes, release of inflammatory mediators by CNS glial and peripheral immune cells, and development of extracellular amyloid-β (Aβ) fibrils that deposit in the brain as amyloid plaques, and impair synaptic plasticity [98]. At the molecular level, the extracellular deposition of insoluble amyloid-β Aβ peptide plaques (39–43 amino acids produced by amyloid precursor protein, APP, peptides) occurs, which can interact with regulatory proteins to phosphorylate the microtubule-associated protein tau. The increase of phosphorylated tau protein (P-S396-tau, P-T181-tau) and Aβ42 detected in plasma EVs is associated with increased risk for AD [98]. Of note, both Aβ and tau are secreted by neurons through the release of EVs [12,26,36,56,90]. Moreover, some authors also studied molecules found in EVs from patients suffering from mild cognitive impairment. Their memory has deficits that do not significantly affect daily functioning, but are often regarded and treated as early phase AD [52,55,99].

Parkinson’s disease (PD) is other common neurodegenerative disorder. Although PD can be described more correctly as a syndrome caused by different genetic and epigenetic alterations, a shared downstream result is the degeneration of dopamine-releasing axon terminals in the striatum and of corresponding neurons in the substantia nigra, which leads to impairment of motor and speech skills [100]. EVs have also been used as a diagnostic platform for PD. Indeed, increased α-synuclein levels in plasma EVs were associated with PD, and disease clinical severity, although results are inconsistent [18,72,88]. Moreover, reduced levels of CSF’s EVs containing apolipoprotein A1 were associated with a higher risk of PD [21].

Multiple sclerosis (MS) and amyotrophic lateral sclerosis (ALS) are very different demyelinating diseases. MS is an autoimmune disease that affects the myelin sheath, which insulates nerve cell fibers in the brain and spinal cord [101], whereas ALS is a motor neuron disease that mainly affects the brain and spinal cord motor neuron cells [102]. For both diseases, several efforts have been made to find disease-related biomarkers in EVs isolated from patients’ biological fluids, with a special emphasis in non-coding RNAs.

Non-coding RNAs (ncRNAs) are also generally found in circulating EVs. There are several ncRNA categories, commonly classified according to their size: the long ncRNAs (lncRNAs) with more than 200 nt, and the small ncRNAs (sncRNAs), including microRNAs (miRs), which present less than 200 nt [103,104]. These ncRNAs have been studied as specific disease “signatures” found in EVs in various disorders including MS/ALS. In fact, for all major group of CNS-related diseases mentioned in this review, published data on EVs ncRNAs have been assessed. For instance, in a recent study by Ebrahimkhani S. et al., the utility of serum exosome miRs as disease biomarkers of MS patients under treatment with fingolimod was assessed, and they found that several combinations of two or three miRNAs could discriminate active from quiescent disease with more than 90% accuracy [79]. Likewise, for ALS, Banack et al. found that eight miRNAs, isolated from neural-enriched EVs, significantly distinguished ALS patients from controls, and thus might assist in early diagnosis of this disease [75].

Gliomas are glial cell-derived brain tumors, classified according to cell type, including ependymomas (ependymal cells), oligodendrogliomas (oligodendrocytes) and astrocytomas (astrocytes). The most common and malignant primary brain tumor, the grade IV astrocytoma/glioblastoma multiform belongs to this group of tumors [105]. Glioblastoma-specific or glioblastoma-rich protein and genetic material can be detected in EVs isolated from the biological fluids of glioblastoma’s patients. The usefulness of using EVs as platforms for the analysis of specific biomarkers, which can help predict the disease outcome and prognosis of GM patients, have been recently established. For example, EGFRvIII is the oncogenic form of epidermal growth factor receptor (EGFR), never found in normal tissues. According to various reports, EGFRvIII protein, mRNA and DNA have been detected in plasma EVs isolated from patients with glioblastoma carrying EGFRvIII, but not in EVs isolated from noncancer patients [28,41,44]. Similarly, multiple studies have also shown that miR-21 levels are higher in EVs isolated from the serum and CSF of patients with glioblastoma. This finding is supported by the higher levels of miR-21 also described in clinical glioblastoma specimens [20,27,46].

Traumatic brain injury (TBI), also known as intracranial injury, is a brain damage caused by external forces [106]. TBI classification can be based on severity (from mild to severe TBI), mechanism (closed or penetrating head injury), or other characteristics (for example, occurring in a specific location or widespread area). TBI usually causes neurological sequels, which can be seen even in mild TBI. Mild TBI may lead to acute symptoms, including chronic traumatic encephalopathy, cognitive impairment, dementia, movement disorders, and motor neuron dysfunction [107]. One of the main clinical challenges in TBI is to accurately identify its occurrence, and to determine the extent of CNS damage. Peltz et al. showed that in war veterans with a history of TBI, CNS-enriched exosome concentration of pTAU, NfL, IL-6, TNFa are associated with cognitive impairment, which may assist clinicians in choosing a more appropriate treatment schedule and follow-up [89].

Psychiatric disorders, like schizophrenia (SCZ) or chronic depression, are estimated to affect a major percentage of the world population and are very difficult to diagnose and manage. Schizophrenia is a neurological disorder characterized by behavioral deficits, associated with impaired locomotor activity and cognitive defects. The diagnosis of schizophrenia is associated with demonstrable alterations in brain structure and changes in dopamine and glutamate neurotransmission in the cortex [108]. In this field, there is a demand for the discovery of biomarkers, which, if successful, can support clinicians in personalized treatment strategies. In this context, Du Y. et al. showed that the expression levels of specific miRs in EVs isolated from the blood of SCZ patients were sensitive to long-term medication [58].

Stroke, which can be categorized into ischemic or hemorrhagic stroke, affects 13.7 million people worldwide each year, and is the second leading cause of death, with 5.5 million deaths each year [109]. A stroke occurs when the blood supply to part of the brain is interrupted or reduced, limiting brain tissue from obtaining oxygen and nutrients, and generally causing brain damage [110]. Several authors explored the importance of the innate immune response as a contributor to the inflammatory response after stroke [111,112,113]. In this regard, Kerr et al. used a technique designated Simple Plex assay (an immunoassay in a microfluidic cartridge) to determine the presence and concentration of some inflammasome proteins in serum EVs of stroke patients [38]. The authors found that protein levels of ASC (caspase-recruitment domain) remained higher in serum-derived EVs from stroke samples, when compared to controls, and performed well as a potential biomarker for this pathological condition [38].

Major depressive disorder (MDD) is a debilitating mental disease characterized by persistent low mood. It affects the behavior as well as various physical functions, such as appetite and sleep [114]. Although the understanding of the neurobiology of MDD has improved in recent years, the knowledge of the mechanisms that may explain the most relevant aspects of the disease is limited. Nonetheless, MDD was associated with smaller hippocampal volumes, changes in activation or connectivity of neural networks, and also changes in the main neurobiological systems that mediate the stress response, including the hypothalamic–pituitary–adrenal (HPA) axis, autonomic nervous system, and immune system [115,116]. The identification of susceptibility biomarkers that might identify individuals prone to depression and disease recurrence would improve treatment and allow for recurrence prevention. A series of studies by different research teams have implicated a systemic metabolic dysfunction known as insulin resistance in the pathophysiology and treatment of disorders of mood and cognition, including MDD [117,118]. Therefore, the insulin-receptor (IR) investigated in these studies was identified in brain areas associated with mood and cognition [119]. Indeed, Nasca et al. recently found that insulin-receptor substrate-1 (IRS-1) was highly abundant in neuronal-derived EVs isolated from plasma samples of MDD patients, compared to nonpathological controls, and that higher IRS-1 levels were associated with suicidality and anhedonia in those individuals. This data could lead to an improved strategy in the treatment of MDD patients [86].

## 5. Future Perspectives and Conclusions

Research based on EVs, specifically neuronal-derived EVs, is rapidly moving forward. We have now recognized a wide range of biological processes mediated by these molecules, and its importance into cell–cell communication, as well as disease spread within the CNS. With increasing knowledge about EVs from specific neurological diseases and the advances in technologies used to analyze these nanostructures, research is now moving towards clinical translation for biomarker platforms. For this, established and validated biomarkers are needed. Indeed, various single reports based on small cohorts have been published, but very few were validated by larger, cross-sectional investigations. The promising results so far point towards a future where brain-derived EVs could be used for not only for diagnosis of CNS-related disorders, but also for patient monitoring, and for studying the influence of the release of this specific EVs in behavioral and mental health related conditions.

Overall, EVs produced in the brain and circulating in peripheral fluids have become an incredible window to the brain, which is expected to assess the disease status of a given patient with great accuracy and simplicity. In the near future, one can anticipate that a relevant biomarker platform may move quickly into clinical context, to provide an enormous amount of information that may assist in clinical patients’ follow-up. This may occur at the physician’s office, as many technological solutions exist today to monitor such biomarkers using point-of-care devices. In a long-term perspective, one may anticipate that the information deriving from EVs may also act as an early disease warning system. It is only natural that the biological/biochemical information of such circulating EVs shall be better and more significant in time, considering the huge developments expected in the fields of nanotechnology, bioengineering and artificial intelligence. Specifically, when these are combined for the same purpose, even lower concentrations of a given pool of biomarkers in very complex samples might be detected. Finally, it will be indeed a breakthrough when such advanced biomarker panel from circulating EVs (that may well derive from CNS) becomes a tool to prevent disease progression.

## Figures and Tables

**Table 1:** Extracellular vesicles protein and protein-coding genes transcripts as biomarkers associated with central nervous system pathological conditions.

Molecule	Disease	Sample Type	Sample Grouping and Size	Patients Gender and Mean Age	EVs Isolation Method	Key Findings	Ref.
pSer312, p-panTyr-IRS-1	AD	Plasma	Patients (N = 24)	14♀ + 10♂ (73 yrs)	Thromboplastin-D + ExoQuick^®^ (System Biosciences) + L1CAM IP	Markers of brain insulin resistance in NDEVs associate with atrophy in AD.	[29]
NPTX2, NRXN2a, AMPA4, NLGN1	AD	Plasma	C.S.S.: Patients (N = 28), HC (N = 28); L.S.: Patients (N = 18 + 18), HC (N = 18)	C.S.S.: Patients and HC—16♀ + 12♂ (73 yrs); L.S.: Patients and HC—8♀ + 10♂ (69 & 78 yrs)	Thromboplastin-D + 3000× *g* (30′ at 4 °C) + ExoQuick^®^ + L1CAM IP	Reduction of the marker levels in NDEVs may be indicative of the extent of cognitive loss and reflect progression of the severity of AD.	[35]
Tau	AD	Plasma	AD patients (N = 20), MCI patients (N = 10), HC (N = 10)	AD—11♀ + 9♂ (75 yrs) MCI—5♀ + 5♂ (76 yrs) HC—7♀ + 3♂ (76 yrs)	Thrombin + 6000× *g* (20′ at 4 °C) + ExoQuick^®^ + L1CAM IP	Tau was found free-floating with a small component inside EVs; full-length Tau was higher inside EVs than in free solution.	[36]
SNAP-25	AD	Serum	AD patients (N = 24), HC (N = 17)	AD—16♀ + 8♂ (78 yrs) HC—13♀ + 4♂ (77 yrs)	10,000× *g* (10′ at RT) + ExoQuick^®^ + L1CAM IP	The levels of SNAP-25 carried by NDEVs were reduced in AD patients (sensitivity 87.5%, specificity 70.6%).	[53]
N-123 tau, N-224 tau	AD	Serum	Patients w/CSF+ biomarkers (N = 4), patients w/CSF- biomarkers (N = 4)	n.m.	4000× *g* (20′ at 4 °C) + ExoQuick^®^ + L1CAM IP	N-224 tau was present in NDEVs, while N-123 tau showed comparable concentrations in both NDEVs and peripherally derived EVs.	[56]
BACE1-AS	AD	Plasma	AD patients (N = 72), HC (N = 62)	AD—38♀ + 34♂ (74 yrs) HC—39♀ + 23♂ (72 yrs)	Thrombin + 14,000× *g* (5′ at 4 °C) + 3000× *g* (15′ at 4 °C) + ExoQuick^®^	EVs BACE1-AS transcript levels in AD patients were significantly higher compared with the HC (sensitivity 87.5%, specificity 61.3%)	[96]
SYP, SYNPO, SYT2, NRGN, GAP43, SYN1	AD and FTD	Plasma	C.S.S.: AD patients (N = 12), FTD patients (N = 16), HC (N = 28); L.S.: AD patients (N = 9), FTD patients (N = 10), HC (N = 19)	C.S.S.: AD—6♀ + 6♂ (74 yrs) FTD—4♀ + 12♂ (64 yrs) L.S.: AD—7♀ + 2♂ (82 yrs) FTD—5♀ + 5♂ (63 yrs)	Thromboplastin-D + 3000× *g* (30′ at 4 °C) + ExoQuick^®^ + L1CAM IP	SYP, SYNPO, SYT2, and NRGN levels were significantly lower in patients with FTD and AD than in HC. Some markers were decreased years before dementia in FTD and AD patients.	[22]
Tau	AD and PD	Plasma	AD patients (N = 106), PD patients (N = 91), HC (N = 106)	AD—49♀ + 57♂ (70 yrs) PD—26♀ + 65♂ (65 yrs) HC—48♀ + 58♂ (67 yrs)	2000× *g* (15′) + ultracentrifugation + L1CAM IP	Tau was significantly higher in PD patients than HCs, but not in AD patients, and correlated with CSF tau.	[25]
pS1292-LRRK2	PD	CSF	PD patients: LRRK2+ mutation (N = 19), LRRK2− (N = 19); HC: LRRK2+ (N = 39), LRRK2- (N = 5)	PD patients: LRRK2+ 16♀ + 3♂ (57 yrs) LRRK2− 8♀ + 11♂ (60 yrs) HC: LRRK2+ 26♀ + 13♂ (63 yrs) LRRK2- 5♀ (60 yrs)	10,000× *g* (30′ at 4 °C) + ultracentrifugation	pS1292-LRRK2 levels in CSF EVs were near saturated in most subjects, 10-fold higher than in urinary EVs, irrespective of LRRK2 mutation status or PD diagnosis.	[30]
Akt, p-mTOR, p-Tyr-IRS-1	PD	Serum	PD patients (N = 60)	17♀+ 43♂ (60 yrs)	4500× *g* (20′ at 4 °C) + ExoQuick^®^ + L1CAM IP	Exenatide-treated patients had elevated expression of tyrosine phosphorylation of IRS-1 and of downstream targets, total Akt and p-mTOR.	[54]
α-synuclein	PD	Plasma	PD patients (N = 267), HC (N = 215)	PD—119♀ + 145♂ (66 yrs) HC—99♀ + 116♂ (66 yrs)	2000× *g* (15′) + ultracentrifugation + L1CAM IP	Levels of α-synuclein in EVs were substantially higher in PD patients than in HC. A significant correlation between α-synuclein found on EVs and disease severity was observed.	[18]
α-synuclein	PD	Serum	PD patients: Tremor type (N = 22), Non-tremor (N = 16); essential tremor (ET) patients (N = 21); HC (N = 18)	Tremor type—10♀ + 12♂ (63 yrs) Non-tremor type—9♀ + 9♂ (62 yrs) ET—10♀ + 11♂ (62 yrs) HC—10♀ + 8♂ (63 yrs)	3000× *g* for (15′ at 4 °C) + ExoQuick^®^ + L1CAM IP	α-synuclein levels were lower in the PD group than in the ET and HC. Levels were lower in the NTD group than in the TD group. α-synuclein was found to moderately aid in PD diagnosis (AUC = 0.675) and had a potential to diagnose NTD (AUC = 0.761).	[72]
α-synuclein	PD	Plasma	Early-stage PD patients (N = 36), Advanced PD patients (N = 17), iRBD patients (N = 20), HC (N = 21)	Early-stage PD—18♀ + 18♂ (64 yrs) Advanced PD—10♀ + 7♂ (67 yrs) iRBD—8♀ + 12♂ (63 yrs) HC—10♀ + 11♂ (64 yrs)	2000× *g* (15′) + ultracentrifugation + L1CAM IP	α-synuclein levels in NDEVs were significantly higher in patients with early-stage PD compared with HCs. Longitudinally increased α-synuclein were associated with higher risk for motor symptom progression in PD.	[88]
Aß1-42, p-Tau-S396, NRGN, SYP, SYT1, SYNPO	MCI	Plasma	MCI patients (N = 61), HC (N = 76)	MCI—39♀ + 22♂ (70 yrs) HC—47♀ + 29♂ (68 yrs)	Thrombin + 10,000 rpm (5′) + ExoQuick^®^ + L1CAM IP	NDEVs concentrations of Aβ1-42 were significantly increased while NRGN, synaptophysin, synaptotagmin, and synaptopodin levels were significantly decreased in patients with MCI.	[52]
apoA1, apoE, apoJ, AnnexinV, Aß-42	MCI, AD and PD	CSF	MCI patients (N = 21), AD patients (N = 27), PD patients (N = 28), young HC (N = 15), middle-aged HC (N = 21), older HC (N = 23)	MCI—7♀ + 14♂ (75 yrs) AD—11♀ + 16♂ (69 yrs) PD—12♀ + 12♂ (64 yrs) Young HC—11♀ + 4♂ (28yrs) Middle-aged HC—10♀ + 11♂ (55 yrs) Older HC—12♀ + 11♂ (73yrs)	Flow cytometric assay	ApoE and Aß-42-positive particle concentrations were reduced in middle and older age subjects, whereas apoAI increased with age. ApoAI and annexin V levels were reduced in MCI and/or AD patients vs. HCs.	[21]
GSN, BCHE	DLB	Plasma	DLB patients (N = 19), AD patients (N = 10), HC (N = 20)	DLB—ratio ♀/♂ 2:3 (72 yrs) AD—ratio ♀/♂ 2:3 (74 yrs) HC—ratio ♀/♂ 2:1 (69 yrs)	2500× *g* (15′) + 16,000× *g* (10′) + SEC	Gelsolin decreased levels were found on EVs from DLB patients, compared to HCs and to AD patients.	[59]
P-tau, Aß1-42, NRGN, REST	MCI and AD	Plasma	MCI patients (N = 20), AD patients (N = 10), MCI-to-AD (ADC) patients (N = 20) HC (N = 10)	MCI—7♀ + 13♂ (69 yrs) ADC—9♀ + 11♂ (75 yrs) AD and HC—n.m.	Thromboplastin-D + ExoQuick^®^ + L1CAM IP	Abnormal NDEVs levels of P-tau, Aß1-42, NRGN and REST accurately predicted conversion of MCI to AD dementia.	[26]
Tau, APP, pTau-T181, Aβ42	MCI and AD	Plasma	MCI patients (N = 12), mild AD patients (N = 12), moderate AD patients (N = 12), severe AD patients (N = 20), HC (N = 12)	MCI—11♀ + 1♂ (75 yrs) Mild AD—11♀ + 1♂ (76yrs) Moderate AD patients 8♀ + 4♂ (79 yrs) Severe AD patients 10♀ + 2♂ (83 yrs) HC—9♀ + 3♂ (69 yrs)	2000× *g* (20′) + 10,000× *g* (20′) + Total Exosome Isolation reagent (Invitrogen^TM^)	Abnormal APP levels and pTau-T181/tTau ratio in EVs demonstrated a high accuracy to define MCI and AD staging.	[90]
FN1, GFAP	NMOSD	CSF	MS patients (N = 10), NMOSD patients (N = 10), idiopathic longitudinally extensive transverse myelitis patients (N = 12)	MS—7♀ + 3♂ (n.m.) NMO—9♀ + 1♂ (n.m.) I-LETM—1♀ + 11♂ (n.m.)	18,000× *g* (30′) + ultracentrifugation	442 significant proteins generated a list of signature molecules of diseases validated primarily by the identification of known markers such as GFAP and FN1, specific to NMO and MS.	[24]
KLKB1, APOE	MS	CSF	RRMS patients (N = 4), non-demyelinating controls (N = 3)	n.m.	Exo-Spin^TM^ (Cell Guidance Systems)	Plasma kallikrein and Apolipoprotein-E4 were increased in CSF-EVs compared to CSF.	[31]
MOG	MS	Serum and CSF	RRMS patients (N = 45), secondary progressive MS (SPMS) patients (N = 30), HC (N = 45)	n.m.	ExoQuick^®^	Exosomal content of MOG strongly correlated with disease activity and was highest in RRMS patients in relapse and in SPMS patients.	[34]
ASMase	MS	CSF	MS patients (N = 95), other central neurological disease (C_OND) patients (N = 45), other peripheral neurological disease (P_OND) patients (N = 31)	MCI—55♀ + 40♂ (37yrs) C_OND—29♀ + 16♂ (43yrs) P_OND—13♀ + 18♂ (58yrs)	Flow cytometric assay	A high number of acid sphingomyelinase-enriched EVs correlated to enzymatic activity and to disease severity.	[43]
SOD1, TDP-43, p-TDP-43, FUS	ALS	Plasma	ALS patients (N = 30) HC (N = 30)	ALS—15♀ + 15♂ (71 yrs) HC – n.m.	1600× *g* (20′) + ultracentrifugation	Microvesicles (MVs) and Exosomes (EXOs) size were increased in ALS patients compared to HCs; MVs of ALS patients were enriched with toxic proteins compared to HCs.	[48]
CUEDC2	ALS	CSF	ALS patients (N = 4), HC (N = 4)	ALS—4♂ (58 yrs) HC—4♂ (59 yrs)	2000× *g* (5′ at 4 °C) + 10,000× *g* (20′ at 4 °C) + ExoRNeasy Serum/Plasma Midi Kit (QIAGEN)	By RNA sequencing, several genes, such as CUEDC2, in CSF EVs were suggested to be candidate disease biomarkers for ALS.	[68]
CD14, Cystatin C	CWML/Brain atrophy	Plasma	Manifest vascular disease patients (N = 994)	210♀ + 784♂ (59 yrs)	3000× *g* (15′) + ExoQuick^®^	EV proteins cystatin C and CD14 were related to CWMLs and the progression of brain atrophy in patients with manifest vascular disease.	[16]
Ras-related small GTPase 10, Annexin VII, UCHL1, Claudin-5, NKCC1, AQP4, SYNGR3, Aβ42, P-T181-tau, P-S396-tau, IL-6, PRPc	TBI	Plasma	Acute mild TBI (N = 18), chronic mild TBI (N = 14), HC (N = 21)	Acute mild TBI—12♀ + 6♂ (21 yrs) Chronic mild TBI—3♀ + 11♂ (20 yrs) HC—14♀ + 7♂ (21 yrs)	Thromboplastin-D + 3000× *g* (30′ at 4 °C) + ExoQuick^®^ + L1CAM IP	Increases in NDEV levels of most neurofunctional proteins in acute mild TBI, and elevations of most NDEV neuropathological proteins in chronic and acute mild TBI delineated phase-specificity.	[60]
FLOT1, Arf6, Rab7a	TBI	CSF	Severe TBI patients (N = 17), HC (N = 18)	Severe TBI—2♀ + 15♂ (40 yrs) HC—n.m.	500× *g* (10′ at 4 °C) + 2000× *g* (30′ at 4 °C) + ultracentrifugation	CSF after severe TBI contains Flotillin+ EVs. Unfavorable outcomes included decreasing Arf6 concentrations and a delayed Rab7a concentration increase.	[64]
Aβ42, NRGN	TBI	Plasma	Mild TBI patients (N = 19), HC (N = 20)	Mild TBI—19♂ (22 yrs) HC—20♂ (22 yrs)	Thrombin + 10,000 rpm (5′) + ExoQuick^®^ + L1CAM or GLAST IP	NDEV and ADEV levels of Aβ42 were significantly higher while NDEV and ADEV levels of neurogranin were significantly lower in mild TBI patients compared to HCs.	[74]
Aβ42, P-tau, PRPc, SYNGR3	TBI	Plasma	TBI patients: W/CI (N = 26), W/o CI (N = 21); Controls: W/CI (N = 19), W/o CI (N = 42)	TBI w/CI—26♂ (75 yrs) TBI w/o CI—3♀ + 18♂ (79 yrs) Controls w/CI—1♀ + 18♂ (80 yrs) Controls w/o CI—7♀ + 35♂ (79 yrs)	Thromboplastin-D + 3000× *g* (30′ at 4 °C) + ExoQuick^®^ + L1CAM IP	Aβ42 and P-tau species, and their respective putative receptors, PrPc and synaptogyrin-3, remain elevated for decades after TBI, and may mediate TBI-associated CI.	[81]
Complement effector/regulatory proteins	TBI	Plasma	sTBI patients (N = 24); mtTBI patients: Early (N = 10) and late (N = 15); sTBI controls (N = 12); mtTBI controls: Early (N = 5) and late (N = 5)	sTBI—12♀ + 12♂ (21 yrs) Early mtTBI—3♀ + 7♂ (38 yrs) Late mtTBI—2♀ + 13♂ (77 yrs) sTBI controls—6♀ + 6♂ (22 yrs) Early mtTBI controls—1♀ + 4♂ (38 yrs) Late mtTBI controls—2♀ + 3♂ (75 yrs)	Thromboplastin-D + 3000× *g* (30′ at 4 °C) + ExoQuick^®^ + GLAST IP	TBI increased plasma ADEVs levels of neurotoxic complement proteins.	[82]
NfL	TBI	Plasma	1–2 TBIs patients (N = 94), ≥3 TBIs patients (N = 56), HC (N = 45)	1–2 TBIs—12♀ + 82♂ (38 yrs) ≥3 TBIs—9♀ + 47♂ (37 yrs) HC—7♀ + 38♂ (38 yrs)	Thrombin + 10,000 rpm (5–10′) + ExoQuick^®^	Repetitive mild TBIs were associated with elevated EV levels of NfL, even years following these injuries.	[83]
UCH-L1, GFAP, NfL, Tau	TBI	Serum	TBI patients (N = 21)	3♀ + 18♂ (52 yrs)	3000× *g* (15′ at 4 °C) + ExoQuick^®^	Patients with diffuse injury displayed higher acute EVs NFL and GFAP concentrations than those with focal lesions. EVs UCH-L1 specific profile was associated with early mortality.	[85]
NfL, GFAP, p-Tau, TNFa, IL-6	TBI	Plasma	TBI patients: W/CI (N = 35), W/o CI (N = 30); Controls: W/CI (N = 30), W/o CI (N = 60)	TBI w/CI—35♂ (77 yrs) TBI w/o CI—4♀ + 26♂ (80 yrs) Controls w/CI—3♀ + 27♂ (82 yrs) Controls w/o CI—9♀ + 51♂ (79 yrs)	Thrombin + ExoQuick^®^ + L1CAM IP	All significantly associated biomarkers combined separated TBI w/vs. w/o CI (AUC = 0.85) and CI w/vs. w/o TBI (AUC = 0.88).	[89]
ASC, caspase-1, IL-1β, IL-18	Stroke	Serum	Patients (N = 16), HC (N = 80)	Stroke—n.m. HC—40♀ + 40♂ (n.m.)	2000× *g* (30′) + Total Exosome Isolation reagent/ 3000× *g* (15′) + ExoQuick^®^	The AUC for ASC was 0.99, whereas the AUC for caspase-1, IL-1β, and IL-18 were 0.75, 0.61, and 0.67, respectively, and can act as biomarkers for stroke.	[38]
Tau, p-tau181	Chronic traumatic encephalopathy	CSF	Patients (N = 15), HC (N = 16)	Patients—15♂ (n.m.) HC—16♂ (n.m.)	1200× *g* (20′ at 4 °C) + 10,000× *g* (30′ at 4 °C) + MagCapture^TM^ Exosome Isolation Kit PS (FUJIFILM Wako Pure Chemical Corporation)	T-tau and p-tau181 levels of CSF-derived EV were positively correlated with the t-tau and p-tau181 levels of total CSF in patients, respectively, but not in the HCs.	[67]
TLN1, FLNA, 14-3-3 proteins	ME/CFS	Plasma	ME/CFS patients (N = 99), ICF patients (N = 6), depression patients (N = 8), HC (N = 56)	n.m.	SEC (qEV iZON Science)	Talin-1, filamin-A, and 14-3-3 family proteins were the most abundant proteins in EVs from ME/CFS patients.	[80]
S100a9, S100a7, lTF, DEFA1	ABE	CSF	Moderate ABE patients (N = 10), severe ABE patients (N = 10), HC (N = 10)	Moderate ABE—4♀ + 6♂ (5.7 days) Severe ABE—3♀ + 7♂ (5.5 days) HC—5♀ + 5♂ (6.4 days)	2000× *g* (20′ at 4 °C) + Ribo™ exosome isolation reagent	A total of 291 dysregulated proteins were identified by comparing ABE patients with HCs, by mass spectrometry. S100a9, S100a7, lTF and DEFA1 were further validated.	[95]
α-synuclein, IL-1β	Epilepsy and ADD	Serum	Epilepsy patients (N = 115), ADD patients (N = 10), HC (N = 146)	Epilepsy—47♀ + 68♂ (9 yrs) ADD—7♀ + 3♂ (8 yrs) HC—68♀ + 76♂ (9 yrs)	3000× *g* (15′ at 4 °C) + ExoQuick^®^	α-synuclein levels were significantly increased in children with epilepsy and with ADD of the CNS and correlated with measures of disease severity. IL-1β levels showed significant correlation only with drug resistance in children with epilepsy.	[77]
Phosphatidylserine	SCZ	CSF	SCZ patients (N = 2), HC (N = 14)	SCZ—2♀ (56 yrs) HC—n.m.	2000× *g* (20′ at RT) + 13,000× *g* (2′ at RT) + flow cytometric assay	SCZ patients displayed more phosphatidylserine+ EVs in CSF compared with HCs.	[15]
IL-34, SYP, TNFR1	MDD	Plasma	MDD patients (N = 34), HC (N = 34)	MDD—14♀ + 20♂ (31 yrs) HC—14♀ + 20♂ (n.m.)	“Sandwich” ELISA w/CD81	IL-34/CD81 levels were significantly higher in MDD group compared to HC group. Synaptophysin (SYP), SYP/CD81, and TNFR1/CD81 were positively correlated with severities of depression and/or various subsymptoms.	[39]
IRS-1	MDD	Plasma	MDD patients (N = 64), HC (N = 29)	MDD—40♀ + 24♂ (43 yrs) HC—11♀ + 18♂ (38 yrs)	Thrombin + 4500× *g* (20′ at 4 °C) + ExoQuick^®^ + L1CAM IP	An increased concentration of IRS-1 in EVs of MDD patients was found, as compared with HC. Gender differences were observed for serine-312 phosphorylation of IRS-1 in MDD patients EVs.	[86]
TNFR1, NF-κB	Bipolar disorder	Plasma	Patients: Infliximab-treated (N = 27), placebo (N = 28)	Infliximab—20♀ + 7♂ (44 yrs) Placebo—24♀ + 4♂ (46 yrs)	Thrombin + 4500× *g* (20′ at 4 °C) + ExoQuick^®^ + L1CAM IP	Higher levels of physical abuse were associated with larger biomarker decreases over time. The antidepressant response to infliximab was moderated by TNFR1. In infliximab-treated participants, reductions in TNFR1 levels were associated with improvement in depressive symptoms.	[84]
MGMT, APNG	GM	Serum	GM patients (N = 17), HC (N = 15)	n.m.	1100× *g* (10′) + immunomagnetic exosomal RNA (iMER) platform	EVs mRNA levels of MGMT and APNG correlate well with levels found in parental cells and change considerably during treatment of seven GM patients.	[19]
EGFRvIII mutation	GM	CSF	GM patients (N = 71)	20♀ + 51♂ (61 yrs)	1500× *g* (10′) + ultracentrifugation	EGFRvIII was detected in CSF-derived EVs for 14/23 EGFRvIII tissue+ GM patients. Only one of the 48 EGFRvIII tissue- patients had the EGFRvIII mutation detected in EVs. Sensitivity and specificity of EVs to detect an EGFRvIII-positive GBM was 61% and 98%, respectively.	[28]
EGFRvIII mutation	GM	Serum/plasma	GM patients (N = 13), HC (N = 6)	GM—4♀ + 8♂ (63 yrs) HC—n.m.	Microfluidic isolation	The ^EV^HB-Chip achieved 94% tumor-EV specificity. EVs from serum and plasma samples from GM patients had mutant EGFRvIII mRNA.	[44]
PD-L1	GM	Serum/plasma	GM patients (N = 21), HC (N = 5)	n.m.	15,000× *g* (10′) + ultracentrifugation	PD-L1 DNA was present in circulating EVs from GM patients where it correlated with tumor volumes of up to 60cm^3^.	[45]
IFN-γ, IL-10, IL-3, B7-1, B7-2, ICOSL	GM	Plasma	GM patients (N = 19), HC (N = 19)	GM—6♀ + 13♂ (n.m.) HC—n.m.	3000 rpm (15′) + OptiPrep^TM^ solution (Sigma-Aldrich) + ultracentrifugation	Cytokines and costimulatory molecules were readily detected but appeared globally reduced in GM patients’ EVs.	[57]
GFAP, Tau	GM	Plasma	GM patients (N = 15), HC (N = 8)	n.m.	Dielectrophoretic (DEP) micro-chip device	For GM diagnosis, EV-GFAP reached 93% sensitivity, 38% specificity, and AUC of 0.65; for EV-Tau, 67% sensitivity, 75% specificity and AUC of 0.71 was disclosed.	[66]
PTRF	Glioma	Serum	Glioma patients (N = 36)	n.m.	10,000× *g* (30′) + ultracentrifugation	A positive correlation between tumor grade and PTRF expression was found in both tumor tissues and blood EVs from GM patients. PTRF expression in exosomes isolated from the sera of GM patients was decreased after surgery.	[37]
EGFRvIII mutation	Glioma	Serum	Grade III glioma patients (N = 23), Grade IV glioma patients (N = 73) Other neurological diseases patients (N = 15), HC (N = 50)	Grade III glioma—4♀ + 19♂ (44 yrs) Grade IV glioma—25♀ + 48♂ (53 yrs) Controls—n.m.	600× *g* (10′) + 2000× *g* (20′) + 10,000× *g* (20′) + Total Exosome Isolation Kit	Sensitivity and specificity of EVs EGFRvIII detection assay in serum were 81.58% and 79.31%, respectively. EGFRvIII expression either in EVs or tissue correlated with poor survival.	[41]
EGFR, NLGN3, PTTG1	Glioma	Serum	Glioma patients (N = 23), HC (N = 12)	Glioma—9♀ + 14♂ (52 yrs) HC—3♀ + 9♂ (59 yrs)	2000× *g* (15′) + ultracentrifugation	Protein expression of EGFR in EVs can accurately differentiate high-grade and low-grade glioma patients, and positively correlates with ki-67 labeling index in tumor tissue. NLGN3 and PTTG1 mRNA in EVs were also validated for detecting glioma patients.	[73]
FASN	Glioma	Plasma	Glioma patients (N = 8 + 9), HC (N = 8 + 3)	n.m.	1000× *g* (7′) + 10,000× *g* (30′) + ultracentrifugation	FASN was elevated in CD63+ and CD81+ EVs in glioma patient samples.	[92]
GFAP, Survivin	Glioma	Serum	Glioma patients (N = 8), HC (N = 3)	Glioma—3♀ + 5♂ (52 yrs) HC—n.m.	10,000× *g* (80′at 4 °C) + ultracentrifugation	Patients with longer time to tumor progression exhibited a decrease in CD9+/SVN+ and CD9+/GFAP+/SVN+ EVs immediately following survivin vaccination; whereas, those with early tumor progression had an increase in the same markers, despite anti-survivin immunotherapy.	[97]
PpIX	Glioma	Plasma	Glioma patients (N = 6)	2♀ + 4♂ (59 yrs)	exoEasy Maxi Kit (Qiagen)	Plasma of patients with avidly fluorescent tumors undergoing FGS contain circulating PpIX+ EVs at levels significantly higher than their predosing background, which correlates with enhancing tumor volumes.	[62]
CD63, CD81	Brain tumors (mixed)	Plasma	GM patients (n.m.), anaplastic astrocytoma patients (n.m.), brain metastases patients (n.m.), meningioma patients (n.m), Pituitary adenoma patients (n.m.), epilepsy controls (n.m.), HC (n.m.)	n.m.	15,000× *g* (15′) + ultracentrifugation	EVs with double positive CD63+/CD81+ expression are enriched in cancer cell lines and patient plasma samples.	[69]

Abbreviations: ABE—acute bilirubin encephalopathy; AD—Alzheimer’s disease; ADD—acquired demyelinating disorders; ADEV—astrocyte-derived extracellular vesicles; ALS—amyotrophic lateral sclerosis; CI—cognitive Impairment; CSF—cerebrospinal fluid; CWML—cerebral white matter lesion; DLB—dementia with Lewy bodies; EV—extracellular vesicles; FTD—frontotemporal dementia; GM—glioblastoma multiforme; HC—healthy controls; ICF—idiopathic chronic fatigue; MCI—mild cognitive impairment; MDD—major depressive disorder; ME/CFS—myalgic encephalomyelitis/chronic fatigue syndrome; miR—microRNA; MS—multiple sclerosis; mtTBI—military veterans traumatic brain injury; NDEV—neuron-derived extracellular vesicles; n.m.—not mentioned; NMOSD—neuromyelitis optica spectrum disorders; PD—Parkinson’s disease; RRMS—relapsing-remitting multiple sclerosis; SCZ—schizophrenia; SEC—size exclusion chromatography; sTBI—sports-related traumatic brain injury; TBI—traumatic brain injury; yrs—years.

**Table 2:** Extracellular vesicles non-coding RNAs as biomarkers associated with central nervous system pathological conditions.

Molecule	Disease	Sample Type	Sample Grouping and Size	Patients Gender and Mean Age	EVs Isolation Method	Key Findings	Ref.
miR-16-5p, miR-125b-5p, miR-451a, miR-605-5p	AD	CSF	Young-onset AD (YOAD) Patients (N = 17), Late-onset AD (LOAD) Patients (N = 13) HC (N = 12)	YOAD—10♀ + 7♂ (61 yrs) LOAD—5♀ + 8♂ (76 yrs) HC—7♀ + 5♂ (67 yrs)	3000× *g* (5′) + miRCURY^TM^ Exosome Isolation Kit (Exiqon)	MiR-16-5p, miR-125b-5p, miR-451a, and miR-605-5p were differentially expressed in the EVs of YOAD patients when compared with HC. In LOAD patients, miR-125b-5p, miR-451a, and miR-605-5p were similarly altered in expression, but miR-16-5p showed similar expression to HC.	[42]
miR-27a-3p, miR-30a-5p, miR-34c, piR_019324, piR_019949, piR_020364	AD	CSF	AD patients (N = 42), MCI patients (N = 17), psychiatric and neurological controls (N = 82)	n.m.	3500× *g* (10′ at 4 °C) + 2X 4500× *g* (10′ at 4 °C) + 10,000× *g* (30′ at 4 °C) + ultracentrifugation	A combined signature consisting of three miRNAs and three piRNAs were suitable to detect AD with an AUC of 0.83. The piRNA signature could predict the conversion of MCI patients to AD with an AUC of 0.86. When combining the smallRNA signature with pTau and Aβ 42/40 ratio the AUC reaches 0.98.	[61]
miR-23a-3p, miR-223-3p, miR-190a-5p, miR-100-3p	AD	Plasma	AD patients (N = 40), HC (N = 40)	AD—25♀ + 15♂ (73 yrs) HC—18♀ + 22♂ (67 yrs)	3000× *g* (15′) + Thrombin + 10,000 rpm (5′) + ExoQuick^®^ (System Biosciences) + L1CAM IP	MiR-23a-3p, miR-223- 3p and miR-190a-5p levels in NDEVs from AD patients were significantly upregulated as compared with HCs, whereas miR-100-3p levels were significantly downregulated.	[93]
miR-204-5p, miR-632	FTD	CSF	GeNFI cohort: *GRN*, *C9orf72* and *MAPT* mutation carriers (N = 38), Non-mutation carriers (N = 11); Sporadic disease cohort: FTD patients (N = 11), PPA patients (N = 6), AD patients (N = 13), HC (N = 10)	Mutation carriers—25♀ + 13♂ (54 yrs) Non-mutation carriers—6♀ + 5♂ (47 yrs) FTD—2♀ + 9♂ (67 yrs) PPA—2♀ + 4♂ (66yrs) AD—5♀ + 8♂ (63 yrs) HC—5♀ + 5♂ (69 yrs)	10,000× *g* (5′) + miRCURY^TM^ Exosome Isolation Kit	In the GeNFI cohort, miR-204-5p and miR-632 were significantly decreased in symptomatic compared with presymptomatic mutation carriers, with an AUC of 0.89 and 0.81, respectively, and 0.93 when combined. In sporadic FTD, only miR-632 was significantly decreased compared with AD and HC (AUC = 0.90).	[47]
miR-233	Dementia	Serum	Dementia patients: First clinic visit AD (ADfirst) (N = 11), Treatment-receiving AD (ADcare) (N = 11), VD (N = 10); HC (N = 16)	ADfirst—5♀ + 6♂ (76 yrs) ADcare—4♀ + 7♂ (79 yrs) VD—4♀ + 6♂ (82 yrs) HC—8♀ + 8♂ (80 yrs)	3000× *g* (15′ at 4 °C) + ExoQuick^®^	The median levels of EVs miR-223 was significantly decreased in dementia patients, when comparing with HC (AUC = 0.875).	[51]
miR-132-3p, miR-212	AD and MCI	Plasma	AD patients (N = 16), AD-MCI patients (N = 16), HC (N = 31)	n.m.	Thrombin + 6000× *g* (20′ at 4 °C) + ExoQuick^®^ + L1CAM IP	Measurement of miR-132-3p in NDEVs showed good sensitivity and specificity to diagnose AD, but did not effectively separate individuals with AD-MCI from HC. MiR-212 was also decreased in NDEVs from AD patients compared to HC.	[55]
let-7e-5p, miR- 125a-5p, miR-23a-3p, miR-375, miR-1468-5p, miR-204-5p, miR-369-5p, miR-423-5p	AD and PD	Plasma	AD patients (N = 5), PD patients (N = 7), HC (N = 34)	AD—18♀ + 22♂ (67 yrs) PD—6♀ + 1♂ (62 yrs) HC—14♀ + 20♂ (33 yrs)	exoRNeasy Serum/Plasma Maxi Kit (QIAGEN) and 8000× *g* (5′) + ExoQuick^®^	Compared to the HC, eight miRNAs were found to be significantly elevated/declined in AD and PD samples, of which fiour miRNAs were newly identified.	[87]
miR-1246, miR-127-3p, miR-19b-3p, miR-134-5p, miR-370- 3p, miR-375, miR-379-5p, miR-382-5p, miR-432-5p, miR-485-5p, miR-493-3p	MS	Serum	RRMS patients (N = 29)	17♀ + 12♂ (34 yrs)	SEC (qEV iZON Science)	Several combinations of two or three miRNAs were able to distinguish active from quiescent disease with greater than 90% accuracy. Additional miRNAs associated with stable remission, and a positive response to fingolimod in patients with active disease prior to treatment.	[79]
miR-9-5p, miR-15a-5p, miR-183-5p, miR- 193a-5p, miR-338-3p, miR-1246	ALS	Plasma	ALS patients (N = 14), HC (N = 8)	ALS—8♀ + 6♂ (62 yrs) HC—n.m.	Vn96 peptide method	MiRNAs with relevance to ALS were found to be deregulated, including miR-9-5p, miR-183-5p, miR-338-3p and miR-1246. MiR-15a-5p and miR-193a-5p were identified for their di- agnostic potential of ALS and association with disability progression, respectively.	[70]
miR-146a-5p, miR-199a-3p, miR-4454, miR-10b-5p, miR-29b-3p, miR-151a-3p, miR-151a-5p, miR-199a-5p	ALS/MND	Plasma	ALS/MND patients (N = 10 + 10), HC (N = 10 + 10)	n.m.	Thrombin + ExoQuick^®^ + L1CAM IP	Five upregulated and three downregulated miRNA sequences significantly distinguished ALS/MND patients from HC in two independent patient cohorts.	[75]
miR-203b-5p, miR-203a-3p, miR-206, miR- 185-5p	TBI	Plasma	TBI patients (N = 16), HC (N = 20)	n.m.	Track Etched Magnetic Nanopore (TENPO) sorting for GluR2	A panel of four miRNAs significantly discriminated TBI patients vs. HC.	[63]
miR-139-5p, miR-18a-5p, miR-103a-3p	TBI	Plasma	1-2 TBIs patients (N = 73), rTBI patients (N = 45), HC (N = 35)	1-2 TBI—7♀ + 66♂ (39 yrs) rTBI—9♀ + 36♂ (41 yrs) HC—4♀ + 31♂ (42 yrs)	3000 rpm (5′) + exoRNeasy Serum/Plasma Kit	MiR-139-5p and miR-18a-5p, were significantly differentially expressed in the rTBI and 1-2 TBI groups. TBI history and neurobehavioral symptom survey scores negatively correlated with miR-103a-3p expression.	[78]
miR-9, miR-124	AIS	Serum	AIS patients (N = 65), HC (N = 66)	AIS—25♀ + 40♂ (64 yrs) HC—30♀ + 36♂ (60 yrs)	21,000× *g* (15′ at 4 °C) + ExoQuick^®^	miR-9 and miR-124 were significantly higher in AIS patients vs. HC (AUCs of 0.8026 and 0.6976, respectively).	[23]
miR-21-5p, miR-30a-5p	IS	Plasma	HIS patients (N = 15), AIS patients days 1-3 (N = 33), AIS patients days 3-7 (N = 32), SIS patients (N = 31), RIS patients (N = 32) HC (N = 24)	HIS—5♀ + 10♂ (58 yrs) AIS days 1-3—9♀ + 24♂ (58 yrs) AIS days 3-7—13♀ + 19♂ (58 yrs) SIS—9♀ + 22♂ (62 yrs) RIS—4♀ + 28♂ (62 yrs) HC—6♀ + 18♂ (57 yrs)	16,000× *g* (10′ at 4 °C) + exoRNeasy Serum/Plasma Kit	MiR- 21-5p and miRNA-30a-5p in combination are promising biomarkers for diagnosing IS and distinguishing among HIS, SIS, and RIS, especially miRNA-30a-5p for the diagnosis of the HIS phase.	[50]
miR-122-3p, miR-200a-5p	NMOSD	Serum	NMOSD in relapsing patients (N = 16), NMOSD in remission patients (N = 15), HC (N = 14)	NMOSD relapsing—14♀ + 2♂ (37 yrs) NMOSD remission—13♀ + 2♂ (39 yrs) HC—12♀ + 2♂ (35 yrs)	Ribo^TM^ Exosome Isolation Reagent (RiboBio)	MiR-122-3p and miR-200a-5p could distinguish NMOSD status, and were significantly upregulated in the serum EVs of relapsing NMOSD compared with that in remitting NMOSD. The two miRNAs had positive correlations with disease severity in NMOSD patients.	[76]
miR-3613-5p, miR-4668-5p, miR-8071, miR-197-5p, miR-4322, miR-6781-5p	mTLE-HS	Plasma	mTLE-HS patients (N = 40), HC (N = 40)	mTLE-HS—15♀ + 25♂ (27 yrs) HC—n.m.	2000× *g* (20′) + 10,000× *g* (20′) + ExoQuick^®^	Among six candidate microRNAs, miR-8071 had the best diagnostic value for mTLE-HS with 83.33% sensitivity and 96.67% specificity, and was associated with seizure severity.	[32]
miR-206, miR619-5p, miR-133a-3p, miR-143-3p, miR-144-5p, miR-499a-5p, miR-3614-5p, miR-941, miR-30c-5p, miR-339-5p, miR-30b-5p, miR-6515-5p	SCZ	Serum	SCZ patients (N = 100), HC (N = 100)	SCZ—50♀ + 50♂ (30 yrs) HC—42♀ + 58♂ (29 yrs)	SEC (qEV iZON Science)	MiR-206 was the most upregulated miRNA in the EVs of SCZ patients. A signature of 11 miRNAs were identified in EVs from SCZ patients and were used to classify samples from SCZ and HC subjects with high accuracy.	[58]
miR-203a-3p	PTSD	Plasma	Discovery set: PTSD patients (N = 12), HC (N = 12); validation set: PTSD patients (N = 10), HC (N = 10)	Discovery set: PTSD—12♂ (31 yrs) HC—12♂ (34 yrs) validation set: PTSD—10♂ (31 yrs) HC—10♂ (31 yrs)	10,000× *g* (10′) + SEC (iZON Science)	The concentration changes of miR-203a-3p in EV and miR-339-5p in EV-depleted plasma were confirmed two independent cohort veterans with PTSD.	[65]
RNU6-1, miR-320, miR-574	GM	Serum	Training set: GM patients (N = 25), HC (N = 25); validation set: GM patients (N = 50), HC (N = 30)	Training set: GM—11♀ + 14♂ (60 yrs) HC—11♀ + 14♂ (60 yrs) validation set: GM—20♀ + 30♂ (61 yrs) HC—16♀ + 14♂ (54 yrs)	ExoQuick^®^	The expression levels of the sncRNA RNU6-1, miR-320 and miR-574-3p were significantly associated with a GM diagnosis. RNU6-1 was consistently an independent predictor of a GBM diagnosis.	[17]
miR-21, miR-27b, miR-130b, miR-193b, miR-218, miR-331, miR-374a, miR-520f, miR-548c	GM	CSF	Discovery cohort 1 patients (N = 24) and HC (N = 15); discovery cohort 2 patients (N = 40) and HC (N = 27); discovery cohort 3 patients (N = 13) and HC (N = 19); validation cohort 4 patients (N = 10) and HC (N = 12); validation cohort 5 patients (N = 18) and HC (N = 20)	Discovery cohort 1—17♀ + 22♂ (61 yrs) discovery cohort 2—32♀ + 35♂ (59 yrs) discovery cohort 3—13♀ + 19♂ (57 yrs) validation cohort 4—5♀ + 17♂ (54 yrs) validation cohort 5—25♀ + 15♂ (58 yrs)	2000× *g* (20′) + ultracentrifugation	Comparison of miRNA profiles between GM patients and HC yielded a tumor “signature” consisting of nine miRNAs, which correlated with GM tumor volume.	[27]
HOTAIR	GM	Serum	GM patients (N = 43), HC (N = 40)	n.m.	Total Exosome Isolation Kit (Invitrogen^TM^)	HOTAIR was present in whole serum and purified EVs but not in serum supernatant depleted of EVs, in GM patients.	[49]
RNU6-1	GM	Serum	GM patients (N = 18), subacute stroke patients (N = 30), acute/subacute hemorrhage patients (N = 30), MS patients (N = 18), brain metastases patients (N = 21), PCNSL patients (N = 12), HC (n = 30),	GM—8♀ + 10♂ (63 yrs) stroke—11♀ + 19♂ (71 yrs) hemorrhage—9♀ + 21♂ (66 yrs) MS—13♀ + 6♂ (41 yrs) PCNSL—4♀ + 8♂ (66 yrs) metastases—10♀ + 11♂ (60 yrs) HC—17♀ + 13♂ (47 yrs)	ExoQuick^®^	RNU6-1 expression was significantly higher in GM patients vs. HC, and also when comparing with patients with non-neoplastic lesions. No significant differences were found between GM patients and brain metastases.	[91]
miR-21	Glioma	CSF	Glioma patients (N = 70), non-glioma controls (N = 25)	Glioma—28♀ + 42♂ (50 yrs) non-glioma—7♀ + 18♂ (54 yrs)	2000× *g* (30′) + 12,000× *g* (25′) + ultracentrifugation	MiR-21 levels in CSF-EVs of glioma patients were found significantly higher than in non-glioma controls; whereas no difference was detected in serum-derived EVs. The CSF-EVs miR-21 levels correlated with tumor spinal/ventricle metastasis and the recurrence with anatomical site preference.	[20]
miR-4443, miR-422a, miR-494-3p, miR-502-5p, miR-520f-3p, miR-549a	Glioma	Serum	Glioma patients (N = 28), HC (N = 8)	Glioma—13♀ + 15♂ (49 yrs) HC—n.m.	3 × 3500 rpm (20′) + ExoQuick^®^	Six overexpressed miRNAs were found on EVs from glioma patients vs. HC. MiR-549a and miR-502-5p expression predicted prognosis in glioma patients.	[33]
miR-301a	Glioma	Serum	Glioma patients (N = 60), HC (N = 43)	Glioma—33♀ + 27♂ (n.m.) HC—n.m.	3000× *g* (15′ at 4 °C) + ExoQuick^®^	MiR-301a levels on EVs were upregulated in glioma patients compared to HC, and correlated with ascending pathological grades. MiR-301a levels were significantly reduced after surgical resection of primary tumors and increased again during GM recurrence, and were independently associated with overall survival.	[40]
miR-21, miR-222, miR-124-3p	Glioma	Serum	Glioma patients (N = 100), brain non-glial metastases patients (N = 11), HC (N = 30)	Glioma—40♀ + 60♂ (n.m.) non-glial metastases—40♀ + 60♂ (n.m.) HC—8♀ + 3♂ (41 yrs)	3000× *g* (15′) + ExoQuick^®^	The expression levels of miR-21, miR-222 and miR-124-3p in EVs of patients with high grade gliomas were significantly higher than those of low grade gliomas and HC, and were decreased in samples obtained after surgery.	[46]
miR-454-3p	Glioma	Serum	Glioma patients (N = 24), HC (N = 24)	n.m.	Ribo^TM^ Exosome Isolation Reagent	MiR-454-3p was significantly downregulated in tumor tissues, while it was upregulated in EVs from the same patients with glioma, corresponding to an AUC of 0.8663. MiR-454-3p expression was lower in the post-operative samples. High miR- 454-3p expression in EVs or low expression in tissues was associated with poor prognosis	[71]
miR-210, miR-5194, miR-449	Glioma	Plasma	GM patients (N = 25), LGA patients (N = 25), Head trauma patients (N = 15)	GM—6♀ + 19♂ (n.m.) LGA—10♀ + 15♂ (n.m.) trauma—6♀ + 9♂ (n.m.)	n.m.	MiR-210 was upregulated in GM and LGA, whereas miR-185, miR-5194, and miR-449 were downregulated in GM and LGA compared to trauma patients. MiR-5194 and miR-449 were significantly decreased in GM patients compared with LGA.	[94]

Abbreviations: AD—Alzheimer’s disease; AIS—acute phase ischemic stroke; ALS—amyotrophic lateral sclerosis; CSF—cerebrospinal fluid; EV—extracellular vesicles; FTD—frontotemporal dementia; GM—glioblastoma multiforme; HC—healthy controls; HIS—hyperacute phase Ischemic stroke; IS—ischemic stroke; LGA—low-grade astrocytoma; MCI—mild cognitive impairment; miR—microRNA; MND—motor neuron disease; MS—multiple sclerosis; mTLE-HS—mesial temporal lobe epilepsy with hippocampal sclerosis; NDEV—neuron-derived extracellular vesicles; n.m.—not mentioned; NMOSD—Neuromyelitis optica spectrum disorders; PCNSL—primary central nervous system lymphoma; PD—Parkinson’s disease; PPA—primary progressive aphasia; PTSD—post-traumatic stress disorder; RIS—recovery phase ischemic stroke; RRMS—relapsing-remitting multiple sclerosis; rTBI—repetitive traumatic brain injury; SCZ—schizophrenia; SEC—size exclusion chromatography; SIS—subacute phase ischemic stroke; TBI—traumatic brain injury; VD—vascular dementia; yrs—years.

## Data Availability

Data sharing not applicable.
